# Effect of drain omission after mastectomy on cosmesis, patient satisfaction and interval to adjuvant therapy

**DOI:** 10.1308/rcsann.2024.0104

**Published:** 2025-04-08

**Authors:** LR Hector, N To, AE Leusink, D Elfadl, V Voynov, N Roche, JE Rusby

**Affiliations:** ^1^ Cambridge University Hospitals NHS Foundation Trust, UK; ^2^ The Royal Marsden NHS Foundation Trust, UK; ^3^ Institute of Immunology and Immunotherapy, University of Birmingham, UK; ^4^ The Institute of Cancer Research, UK; ^5^ Chelsea and Westminster Hospital NHS Foundation Trust, UK; ^6^ The Dudley Group NHS Foundation Trust, UK

**Keywords:** Mastectomy, Simple, Drains, Patient satisfaction, Adjuvant treatment

## Abstract

**Introduction:**

Omission of closed suction drains in women undergoing simple mastectomy has become the standard in the United Kingdom (UK) with studies demonstrating no difference in symptomatic seroma rates or complications. A theoretical concern is that a large-volume seroma distorts the skin envelope, potentially resulting in inferior long-term postoperative aesthetic appearance and patient satisfaction. Furthermore, the seroma may lead to a delay in adjuvant treatment, in particular, chest wall radiotherapy. There is currently no objective scoring system to evaluate the postoperative appearance after simple mastectomy.

**Methods:**

Patients who had undergone a drainless unilateral simple mastectomy at the Royal Marsden Hospital attending for annual surveillance contralateral mammography between October 2016 and July 2017 were invited to complete a BREAST-Q questionnaire and attend medical photography for panel assessment of aesthetic outcome. Patient satisfaction in this cohort was compared with results from the UK National Mastectomy and Breast Reconstruction Audit (NMBRA) 2011, which was conducted at a time when surgical drains were routinely placed.

**Results:**

The proportion of patients satisfied with their appearance was similar to that of NMBRA 2011. BREAST-Q results were in line with the published literature. A panel assessment scoring system for simple mastectomies was developed. There was no difference in delays to adjuvant treatment between the study and NMBRA cohort.

**Conclusions:**

Omission of drains in women undergoing simple mastectomy did not result in inferior aesthetic outcomes or lower patient satisfaction, nor did it result in delay to adjuvant treatment. BREAST-Q results were in line with the literature. A panel assessment scoring system for simple mastectomy was developed.

## Introduction

Breast cancer is the most common cancer in the United Kingdom (UK) with more than 55,000 cases diagnosed annually.^1^ Surgery is performed in more than 80% of breast cancer cases and approximately 43% of these require a mastectomy. In the UK, immediate breast reconstructions are undertaken in 13–36% of cases, which results in approximately 10,000 patients undergoing simple mastectomies without reconstruction per annum.^2^

Postoperative seroma formation is one of the most common sequelae of simple mastectomy, with the reported incidence ranging from 15% to 85%.^3–5^ Surgical drains have traditionally been used to reduce seroma formation and historically patients would remain hospitalised with the drain for up to 7 days. In 2009, the National Health Service (NHS) Improvement Transforming Inpatient Care Programme highlighted the need for enhanced recovery programmes such as the 23-h breast care model, which strongly encouraged a reduction in the use of wound drains.^6^ This is supported by numerous studies that have demonstrated that drains do not reduce the incidence of symptomatic seroma formation and are in fact associated with increased postoperative pain and prolonged hospital stay.^2–10^ Since 2011 the breast surgery unit at the Royal Marsden Hospital (RMH) has gradually adopted a no-drain policy after simple mastectomy, regardless of axillary surgery. Postoperative seromas are not routinely aspirated unless restricting movement or causing pain that is not controlled with simple analgesia, to reduce the risk of introducing infection during aspiration.

Anecdotally, there has been concern among breast surgeons that a significant postoperative seroma may compromise the postoperative cosmetic outcome by expanding the skin envelope and preventing adhesion of the skin to the chest wall. The National Mastectomy and Breast Reconstruction Audit (NMBRA) in 2011 found that although most women (83%) were satisfied with their appearance when clothed following a simple mastectomy, the majority (58%) were unsatisfied when unclothed.^11^ The relationship between patient satisfaction with the post-treatment outcome, psychological wellbeing and improved quality of life following breast cancer has been well documented.^12–14^ Although many studies report both patient-reported and panel assessment of aesthetic outcomes, they focus on patients who have undergone oncoplastic breast surgery rather than simple mastectomy. This is supported by that fact that there are numerous scoring systems of postoperative aesthetic outcome for both breast-conserving surgery (BCS) and breast reconstruction; however, no such scoring systems for simple mastectomies exist.^15–17^

There are no studies reporting on the impact of drain omission on patient-reported and panel assessment of outcomes after simple mastectomy. The James Lind Alliance Priority Setting Partnership featured outcome after mastectomy in priority 8 and 15, recognising the absence of information for patients opting not to have immediate breast reconstruction and the importance of surgical techniques in improving the outcome.^18^

The aim of this study was to assess patient satisfaction with postoperative outcome after drainless simple mastectomy (with or without axillary surgery). Because the change in practice had occurred in the context of a National Improvement Programme, it was not felt appropriate to carry out a randomised controlled trial. Instead, we compared the current results with those from the NMBRA, given that this was carried out at a time when drain use post-mastectomy was universal.^11^ Secondarily, we investigated whether drain omission resulted in delay to adjuvant treatment and assessed the aesthetic outcome of the chest wall by panel assessment of medical photographs, using a scoring system specifically devised for simple mastectomy.

## Methods

### Patient recruitment

Internal institutional ethical approval was obtained from the RMH (reference SE546) because this study was undertaken as a service evaluation of routine care. Patients who had undergone a drainless unilateral simple mastectomy, with or without axillary surgery, at RMH Chelsea or Sutton due for their annual surveillance contralateral mammogram between October 2016 and July 2017 were invited to participate in the study. A study invitation letter, consent form and paper BREAST-Q questionnaire were posted to eligible patients. Once consent was obtained, patients were asked to attend medical photography for two-dimensional photographs and return the completed questionnaires at the same visit as their surveillance mammogram. Patients were excluded if they had received prior radiotherapy, had an intraoperative drain inserted, underwent immediate or delayed breast reconstruction or experienced regional or distant disease recurrence.

### Patient-reported outcome measures

Patient satisfaction was assessed using the BREAST-Q questionnaire simple mastectomy module. The BREAST-Q questionnaire is a well-validated patient-reported outcome tool with eight domains. These include satisfaction with breast (mastectomy site), psychosocial, physical and sexual wellbeing, and four domains relating to satisfaction with information and staff. Most questions are scored on a Likert scale of 1 to 4 and the answers are transformed into a Rasch score from 0 to 100, where 100 is the best possible score.^19,20^ BREAST-Q scores were not reported in the NMBRA 2011; however, participants were asked similar questions with respect to satisfaction and gave a score of 1–4 so answers to this question were comparable with our cohort and provided a benchmark (Table 1).

**Table 1:** NMBRA 2011 questionnaire^11^ (same as BREAST-Q^™^ Breast Cancer Core Scale Version 2.0 Satisfaction with Breasts Domain)

With your breast area in mind, in the past two weeks, how satisfied or dissatisfied have you been with:
How you look in the mirror clothed
How comfortably your bras fit
Being able to wear clothing that is more fitted
How you look in the mirror unclothed

NMBRA = UK National Mastectomy and Breast Reconstruction Audit

### Panel assessment of aesthetic outcome

Two-dimensional anterior and lateral views were assessed by a panel consisting of six breast surgeons, one breast cancer nurse specialist and one prosthetic fitting specialist, all from the same institution. In the absence of any widely used, validated scoring system to assess the postoperative appearance of simple mastectomies, the following scoring system was devised (Table 2). The scoring system consists of three 3-point domains assessing prominence of the mastectomy scar, adherence of the scar to chest wall and the presence of excess skin, dog ears or residual swelling. A 4-point domain assessing the overall appearance was also used to reflect the 4-point scale Harvard scale commonly used in evaluation of BCS.^15^ Panel members assessed and scored images independently and median panel scores and interquartile range (IQR) were calculated.

**Table 2:** Simple mastectomy scoring system

Description	Scale
Prominence of scar Adherence of skin to the chest wal Presence of excess skin, dog ear or swelling	1 = Large 2 = Moderate 3 = None or minimal
Overall score for appearance	1 = Poor 2 = Satisfactory 3 = Good 4 = Excellent

Fleiss’ kappa was calculated to determine the level of agreement between panellists. Kappa results are interpreted as: <0.00, poor agreement; 0.00–0.20, slight agreement; 0.21–0.40, fair agreement; 0.41–0.60, moderate agreement; 0.61–0.80, substantial agreement; and 0.81–1.00, almost perfect agreement.

### Interval to first adjuvant treatment

Descriptive statistics were used to calculate time interval between surgery and first adjuvant treatment (chemotherapy or radiotherapy) for the study cohort and this was compared with time interval of the NMBRA patient cohort from RMH to determine whether drain omission resulted in a delay in receipt of adjuvant treatment.

### Data storage and statistical analysis

Clinicopathological data were collected from the electronic patient records and entered into a secure password-protected Excel spreadsheet along with BREAST-Q and panel assessment scores. Patient images were captured by the RMH medical photographer and stored in the patient’s records. Written informed consent for publication of clinical images was obtained from the patients. A copy of the consent form was available for review by the Editor. The data were analysed using Excel and SPSS (version 29) and are presented using descriptive statistics using mean and standard deviation or median and IQR where required. Student’s *t*-test was applied to determine whether there were statistically significant differences in BREAST-Q scores between participants who attended medical photography compared with those who did not. The coefficient of determination (*R*^2^) was calculated to determine whether a correlation between BREAST-Q scores for patient ‘satisfaction with breasts’ and panel assessment scores exists.

## Results

### Patient characteristics

**Figure 1:**
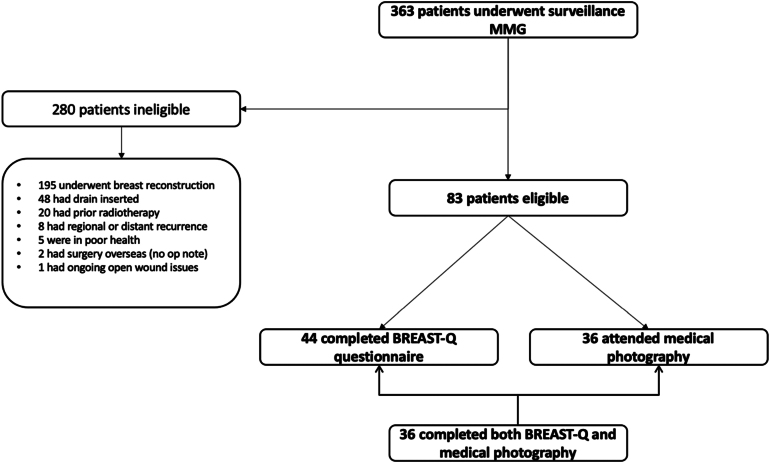
Flow diagram of patient eligibility

Between October 2016 and July 2017 a total of 363 patients underwent contralateral surveillance mammography after unilateral mastectomy across both sites. Of these, 83 patients were deemed eligible and were invited to participate in the study, of whom 44 (53.01%) returned BREAST-Q questionnaires and 36 (43.47%) consented to photography (Figure 1). The median age at the time of surgery was 69.7 years (IQR 58.3–74.6 years) and median body mass index (BMI) was 26.70kg/m^2^ (IQR 23.35–31.15kg/m^2^) (Table 3). The median time from surgery to study participation was 34 months (IQR 23–46 months). Of the 44 women who completed the BREAST-Q questionnaire, 40 (90.9%) had axillary surgery, 29 (65.9%) underwent sentinel lymph node biopsy and 11 (25.0%) underwent axillary lymph node dissection. Twenty-one (47.7%) received radiotherapy, all of whom had chest wall irradiation, six (13.6%) received supraclavicular fossa radiotherapy, one (2.3%) received axillary radiotherapy and one (2.3%) received both. The small sample size precluded analysis for statistical associations between these variables and either the BREAST-Q ‘satisfaction with breasts’ or overall panel score. Six of the 44 patients (13.6%) underwent seroma aspiration, to relieve significant discomfort not alleviated by simple analgesia.

**Table 3:** Participant demographics

Demographics (*N* = 44)	Median (IQR)
Age at time of surgery (years)	69.66 (58.33–74.56)
BMI (kg/m^2^)	26.7 (23.35–31.15)
Ethnicity, *n* (%)	
White	40 (90.9)
Asian	4 (9.1)
Smoking status, *n* (%)	
Never smoked	28 (63.6)
Previous smoker	12 (27.3)
Current smoker	4 (9.1)

BMI = body mass index; IQR = interquartile range

### Patient satisfaction

The proportion of patients who indicated that they were satisfied and very satisfied in the breast satisfaction subset scores in both this study and in the published NMBRA data are compared below (Table 4).^11^

**Table 4:** Comparison of breast satisfaction domain questions for study vs NMBRA results

PROM question	Proportion patients satisfied and very satisfied in study (%)	Proportion patients satisfied and very satisfied in NMBRA (%)
How you look clothed	80.0	83.0
How comfortably bra fits	66.0	73.0
Being able to wear fitted clothing	69.0	65.0
How you look unclothed	43.0	42.0

NMBRA = UK National Mastectomy and Breast Reconstruction Audit; PROM = patient-reported outcome measure

The overall median BREAST-Q scores were 44.0/100 (IQR 36.0–55.0) for satisfaction with breast, 65.0/100 (IQR 49.5–80.5) for psychosocial wellbeing, 42.0/100 (IQR 26.8–63.0) for sexual wellbeing and 71.0/100 (IQR 62.3–82.0) for physical wellbeing (chest).

There was no statistically significant difference in median BREAST-Q scores between patients who completed photography and those who declined photography, except for satisfaction with office staff, which is likely as a result of a small sample size.

### Panel assessment of aesthetic outcomes

**Figure 2:**
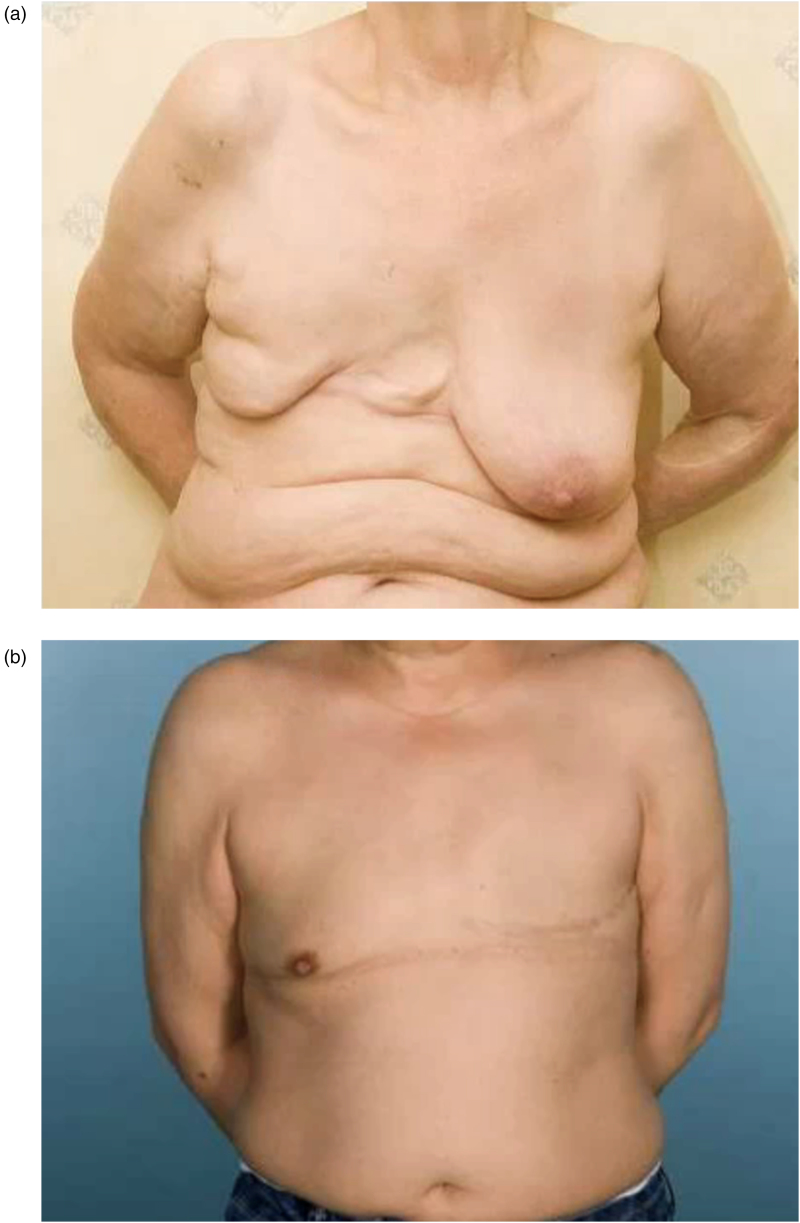
(a) Participant with a low panel score. (b) Participant with a high panel score.

The median panel scores are listed in Table 5. There was fair agreement between panellists when assessing the overall appearance (Fleiss’ kappa 0.23). Examples of participants with high and low panel scores are presented in Figure 2a and 2b, respectively.

**Table 5:** Summary of median panel assessment scores

*n* = 36	Prominence of mastectomy scar (score 1–3)	Adherence of scar to chest wall (score 1–3)	Presence of excess skin/residual swelling (score 1–3)	Overall score (score 1–4)
Median (IQR)	2.73 (2.34–2.91)	2.27 (2.09–2.36)	2.36 (2.09–2.82)	2.72 (2.00–3.23)
Fleiss’ kappa	0.23 (fair)	0.09 (slight)	0.41 (moderate)	0.23 (fair)

IQR = interquartile range

### Correlation between patient satisfaction and panel assessment

**Figure 3:**
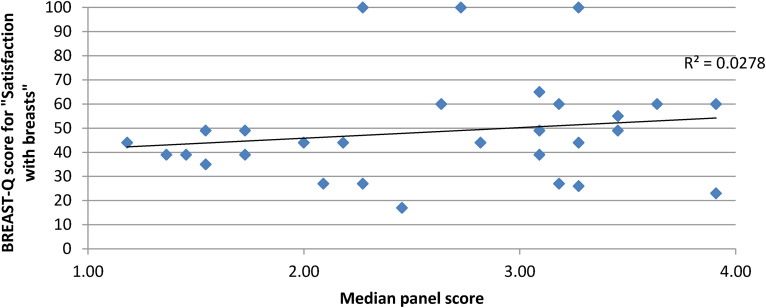
Correlation between panel assessment scores and patient satisfaction scores

There was minimal correlation between overall panel score and patient-reported satisfaction with the breast area, coefficient of determination (*R*^2^) = 0.0278 (Figure 3).

### Interval to first adjuvant treatment

The median time from surgery to adjuvant radiotherapy in the study cohort was 70 days (IQR 57–157) and 75 days (IQR 43–171) in the NMBRA cohort. The median time from surgery to adjuvant chemotherapy was 38 days (IQR 34–47) in the study cohort and 38 days (IQR 32–45) in the NMBRA cohort. The adjuvant therapy characteristics of both cohorts are outlined in Table 6.

**Table 6:** Adjuvant therapy in study vs NMBRA cohort

Type of treatment	Study cohort (*N* = 49)	NMBRA cohort (*N* = 184)
No adjuvant treatment (%)	10 (20.4)	83 (45.1)
Chemotherapy (%)	16 (32.7)	83 (45.1)
Neoadjuvant	6 (37.5)	28 (33.7)
Adjuvant	10 (62.5)	55 (66.3)
Radiotherapy (%)	21 (42.9)	79 (42.9)
Neoadjuvant	0 (0.0)	0 (0.0)
Adjuvant	21 (100)	79 (100)

NMBRA = UK National Mastectomy and Breast Reconstruction Audit

## Discussion

This is the first study to evaluate patient satisfaction and cosmetic outcome in a cohort of patients who have undergone simple mastectomies without closed suction drains.

Drains were traditionally used in simple mastectomies to reduce the risk of large-volume seroma formation postoperatively. The shift towards drainless mastectomy not only reduced patient discomfort and anxiety after surgery, but also reduced the length of hospital stay and increased the utilisation of day case surgery.^21–23^ Drainless-enhanced recovery and early discharge are therefore not only beneficial for the individual patient, but also for the health institution, both in terms of financial and human resources.

One of the remaining concerns regarding drain omission was that the initial formation of a large-volume seroma causes stretching and distortion of the skin envelope, resulting in a suboptimal long-term aesthetic outcome and reduced patient satisfaction with postoperative result. In this study we report a similar level of patient-reported satisfaction with the breast area to that reported in the NMBRA, a cohort of patients who had simple mastectomies with surgical drains, which suggests that drain omission does in fact not have a negative impact on patient satisfaction with the final postoperative aesthetic result.^11^ BREAST-Q scores across all domains were in line with scores cited in the literature, with breast satisfaction scores generally lower in patients who have undergone simple mastectomy than in those who have undergone BCS, implant-based or autologous breast reconstruction.^24–26^

Currently, there is no published scoring system for simple mastectomy. There are, however, numerous published panel assessment scoring systems for both BCS and breast reconstruction. A systematic review by Maass *et al* described 12 scoring systems for breast reconstruction.^27^ None of the scoring systems is widely adopted and none encompassed all seven of the modified Medical Outcomes Trust criteria developed by the Scientific Advisory Committee to determine methodological quality of medical assessment tools.^28^ All of the scoring systems lacked responsiveness and interpretability, which are crucial in ensuring clinical meaningfulness. Only 1 of the 12 scoring systems demonstrated satisfactory validity, although this was limited by a wide range of inter- and intra-rater agreements.^27^

This study reports the first scoring tool to facilitate objective assessment of postoperative appearance after simple mastectomy by panel assessment. Inter-rater agreement ranged from slight to moderate, with fair agreement in the overall appearance domain. This may have been as a result of the variation in panellist expertise and experience, which have been shown to affect inter-rater agreement.^29^ Intra-rater agreement was not assessed. Further work to validate and apply the simple mastectomy panel assessment scoring system more widely is ongoing.

There was no correlation between patient and panel assessment scores in this study. Discordance between patient-reported outcome measures (PROMs) and panel assessment of aesthetic outcome has been consistently reported in the literature, with the latter often being less favourable.^30–34^ Patient opinion of their postoperative outcome may be influenced by external factors such as treatment experience, relationship with healthcare providers and oncological outcome. Patient scoring may also reflect underlying personality differences or acceptance of the diagnosis and emotions associated with this.^35^ The lack of correlation with clinicians’ opinion may reflect a disconnect between what patients value and what clinicians favour, or a clinician’s familiarity with the whole spectrum of possible appearance from the best to the worst and an internalised understanding of the relative complexity of the case that colours the assessment of the outcome, whereas most patients are limited in knowledge to their own experience and possibly that of family members or friends who have experienced similar surgery. Overall, the patient’s own assessment of her outcome is most relevant; however, objective assessment of outcome is also necessary to allow benchmarking and assessment of operative and perioperative techniques.

The time interval to adjuvant treatments was very similar across both cohorts of patients. The median time (IQR) from surgery to adjuvant radiotherapy in the study cohort was 70 days (57–157 days) and 75 days (43–171 days) in the NMBRA cohort. The median time from surgery to adjuvant chemotherapy was 38 days (34–47 days) in the study cohort and 38 days (32–45 days) in the NMBRA cohort. This confirms that drain omission did not result in significant delays to adjuvant treatment.

### Study limitations

This study has several limitations. This study reports findings in a single institution, which may therefore limit generalisability. The small number of patients precluded further statistical analysis of variables associated with patient satisfaction and panel assessment scores. Reasons for the low uptake may include the older age of the simple mastectomy population (mean age 67 years) compared with other series evaluating postoperative appearance. Furthermore, a consistent concern for studies using PROMs and assessing appearance is that women who are more satisfied with their results and give better BREAST-Q and panel assessment results may be more likely to participate than those with poor results. This selection bias may equally apply to this study, although there was no statistically significant difference in scores between patients who attended medical photography and those who did not, suggesting this may not be the case. Panel bias is difficult to eliminate, and although all panellists were from the same institution they were not directly involved in the surgeries and patient images were anonymised to reduce bias as much as possible. Formal cost analysis was also not undertaken as part of this study. There was a difference in duration of follow-up between the study cohort (34 months) and the NMBRA (18 months). In oncoplastic surgery this is relevant because implant reconstructions appear to deteriorate over time and the contralateral breast may also become larger and more ptotic. After unilateral simple mastectomy where symmetry is not relevant, the ipsilateral appearance is not likely to alter much over time.

This study shows that drain omission after simple mastectomy does not impact patient satisfaction with their postoperative appearance. The findings of this study may encourage units still using drains following simple mastectomy to transition towards drain omission.

## Conclusion

This study demonstrates that omission of drains after simple mastectomy does not negatively impact results in terms of patient satisfaction and panel assessment of aesthetic outcome. These results are comparable to those from a large national audit and the literature on patient satisfaction after simple mastectomy. Furthermore, omission of drains did not lead to a difference in delay to adjuvant treatment. This study draws attention to the need to evaluate both subjective (PROMs) and objective assessment of outcome (providing a tool for panel assessment) in a patient cohort that is under-represented in the literature.

## Data Availability

The data sets generated and analysed during the current study are not publicly available but are available from the corresponding author on reasonable request.

## References

[1] Cancer Research UK. Together we are beating cancer. https://www.cancerresearchuk.org/health-professional/cancer-statistics/statistics-by-cancer-type/breast-cancer#heading-Three (cited October 2022).

[2] Jeevan R, Mennie JC, Mohanna PN *et al.* National trends and regional variation in immediate breast reconstruction rates. *Br J Surg* 2016; **103**: 1147–1156. 27324317 10.1002/bjs.10161

[3] Troost MS, Kempees CJ, De Roos MAJ. Breast cancer surgery without drains: no influence on seroma formation. *Int J Surg* 2015; **13**: 170–174. 25486263 10.1016/j.ijsu.2014.11.050

[4] Taylor JC, Rai S, Hoar F *et al.* Breast cancer surgery without suction drainage: the impact of adopting “no drains” policy on symptomatic seroma formation rates. *Eur J Surg Oncol* 2013; **39**: 334–338. 23380200 10.1016/j.ejso.2012.12.022

[5] Boostrom SY, Throckmorton AD, Boughey JC *et al.* Incidence of clinically significant seroma after breast and axillary surgery. *J Am Coll Surg* 2009; **208**: 148–150. 19228516 10.1016/j.jamcollsurg.2008.08.029

[6] NHS Improvement. Transforming inpatient care programme consolidation report - from testing to spread. https://www.england.nhs.uk/improvement-hub/wp-content/uploads/sites/44/2017/11/Transforming-Inpatient-Care-from-Testing-to-Spread.pdf (cited February 2024).

[7] Jain PK, Sowdi R, Anderson ADG, MacFie J. Randomized clinical trial investigating the use of drains and fibrin sealant following surgery for breast cancer. *Br J Surg* 2004; **91**: 54–60. 14716794 10.1002/bjs.4435

[8] Purushotham AD, Mclatchie E, Young D *et al.* Randomized clinical trial of no wound drains and early discharge in the treatment of women with breast cancer. *Br J Surg* 2000; **36**: 286–292. 10.1046/j.0007-1323.2001.02031.x 11872051

[9] Ebner FK, Friedl TWP, Degregorio N *et al.* Does non-placement of a drain in breast surgery increase the rate of complications and revisions? *Geburtshilfe Frauenheilkd* 2013; **73**: 1128–1134. 24771899 10.1055/s-0033-1351071 PMC3862046

[10] Baker E, Piper J. Drainless mastectomy: is it safe and effective? *Surgeon* 2017; **15**: 267–271. 26907221 10.1016/j.surge.2015.12.007

[11] Jeevan R, Cromwell D, Browne J *et al.* National Mastectomy and Breast Reconstruction Audit 2011. https://www.ic.nhs.uk (cited January 2024).

[12] Kim MK, Kim T, Moon HG *et al.* Effect of cosmetic outcome on quality of life after breast cancer surgery. *Eur J Surg Oncol* 2015; **41**: 426–432. 25578249 10.1016/j.ejso.2014.12.002

[13] Al-Ghazal SK, Fallowfield L, Blamey RW. Does cosmetic outcome from treatment of primary breast cancer influence psychosocial morbidity? *Eur J Surg Oncol* 1999; **25**: 571–573. 10556001 10.1053/ejso.1999.0708

[14] Al-Ghazal SK, Sully L, Fallowfield L, Blamey RW. The psychological impact of immediate rather than delayed breast reconstruction. *Eur J Surg Oncol* 2000; **26**: 17–19. 10718173 10.1053/ejso.1999.0733

[15] Harris JR, Levene MB, Svensson G, Hellman S. Analysis of cosmetic results following primary radiation therapy for stages I and II carcinoma of the breast. *Int J Radiat Oncol Biol Phys* 1979; **5**: 257–261. 110740 10.1016/0360-3016(79)90729-6

[16] Godden AR, Wood SH, James SE *et al.* A scoring system for 3D surface images of breast reconstruction developed using the Delphi consensus process. *Eur J Surg Oncol* 2020; **46**: 1580–1587. 32620404 10.1016/j.ejso.2020.05.016 PMC7443694

[17] Visser NJ, Damen THC, Timman R *et al.* Surgical results, aesthetic outcome, and patient satisfaction after microsurgical autologous breast reconstruction following failed implant reconstruction. *Plast Reconstr Surg* 2010; **126**: 26–36. 20595835 10.1097/PRS.0b013e3181da87a6

[18] James Lind Alliance. Breast cancer surgery top 10. https://www.jla.nihr.ac.uk/priority-setting-partnerships/breast-cancer-surgery/top-10-priorities.htm (cited April 2024).

[19] Pusic AL, Klassen AF, Scott AM *et al.* Development of a new patient-reported outcome measure for breast surgery: the BREAST-Q. *Plast Reconstr Surg* 2009; **124**: 345–353. 19644246 10.1097/PRS.0b013e3181aee807

[20] Cano S, Klassen A, Scott A *et al.* The Breast-Q: further validation in independent clinical samples. *Plast Reconstr Surg* 2012; **129**: 293–302. 22286412 10.1097/PRS.0b013e31823aec6b

[21] Saratzis A, Soumian S, Willetts R *et al.* Use of multiple drains after mastectomy is associated with more patient discomfort and longer postoperative stay. *Clin Breast Cancer* 2009; **9**: 243–246. 19933080 10.3816/CBC.2009.n.041

[22] Chadha NK, Cumming S, O’Connor R, Burke M. Is discharge home with drains after breast surgery producing satisfactory outcomes? *Ann R Coll Surg Engl* 2004; **86**: 353–357. 15333173 10.1308/147870804263 PMC1964246

[23] Jackson PC, MacInnes EG, Nicholson JK *et al.* Mastectomy without drains reduces cost with no detriment to patient outcome. *Cureus* 2019; **11**: e5160. 31528512 10.7759/cureus.5160 PMC6743667

[24] Howes BHL, Watson DI, Xu C *et al.* Quality of life following total mastectomy with and without reconstruction versus breast-conserving surgery for breast cancer: a case-controlled cohort study. *J Plast Reconstr Aesthetic Surg* 2016; **69**: 1184–1191. 10.1016/j.bjps.2016.06.004 27406255

[25] Liu T, Freijs C, Klein HJ *et al.* Patients with abdominal-based free flap breast reconstruction a decade after surgery: a comprehensive long-term follow-up study. *J Plast Reconstr Aesthet Surg* 2018; **71**: 1301–1309. 30025757 10.1016/j.bjps.2018.06.009

[26] Eltahir Y, Werners LLCH, Dreise MM *et al.* Quality-of-life outcomes between mastectomy alone and breast reconstruction: comparison of patient-reported BREAST-Q and other health-related quality-of-life measures. *Plast Reconstr Surg* 2013; **132**: 201e–209e. 10.1097/PRS.0b013e31829586a7 23897347

[27] Maass SWMC, Bagher S, Hofer SOP *et al.* Systematic review: aesthetic assessment of breast reconstruction outcomes by healthcare professionals. *Ann Surg Oncol* 2015; **22**: 4305–4316. 25691279 10.1245/s10434-015-4434-2

[28] Lohr KN, Aaronson NK, Alonso J *et al.* Evaluating quality-of-life and health status instruments: developing of scientific review criteria. *Clin Ther* 1996; **18**: 979–992. 8930436 10.1016/s0149-2918(96)80054-3

[29] Potter S, Harcourt D, Cawthorn S *et al.* Assessment of cosmesis after breast reconstruction surgery: A systematic review. *Ann Surg Oncol* 2011; **18**: 813–823. 20972633 10.1245/s10434-010-1368-6

[30] O’Connell RL, Di Micco R, Khabra K *et al.* Comparison of immediate versus delayed DIEP flap reconstruction in women who require postmastectomy radiotherapy. *Plast Reconstr Surg* 2018; **142**: 594–605. 29927832 10.1097/PRS.0000000000004676 PMC6112844

[31] O’Connell RL, DiMicco R, Khabra K *et al.* Initial experience of the BREAST-Q breast-conserving therapy module. *Breast Cancer Res Treat* 2016; **160**: 79–89. 27637781 10.1007/s10549-016-3966-x

[32] Potter S, Thomson H, Greenwood R *et al.* Evaluating the cosmetic outcome of breast reconstruction: a comparison of patients’ and healthcare professionals’ views and their associations with patient satisfaction and quality of life. *Cancer Res* 2009; **69**: 4135.

[33] Heil J, Dahlkamp J, Golatta M *et al.* Aesthetics in breast conserving therapy: do objectively measured results match patients’ evaluations? *Ann Surg Oncol* 2011; **18**: 134–138. 20697820 10.1245/s10434-010-1252-4

[34] Merie R, Browne L, Cardoso JS *et al.* Proposal for a gold standard for cosmetic evaluation after breast conserving therapy: results from the St George and Wollongong Breast Boost trial. *J Med Imaging Radiat Oncol* 2017; **61**: 819–825. 28834326 10.1111/1754-9485.12645

[35] Beesley H, Ullmer H, Holcombe C, Salmon P. How patients evaluate breast reconstruction after mastectomy, and why their evaluation often differs from that of their clinicians. *J Plast Reconstr Aesthet Surg* 2012; **65**: 1064–1071. 22475685 10.1016/j.bjps.2012.03.005

